# Binding Interactions of Zinc Cationic Porphyrin with Duplex DNA: From B-DNA to Z-DNA

**DOI:** 10.3390/ijms19041071

**Published:** 2018-04-04

**Authors:** Tingxiao Qin, Kunhui Liu, Di Song, Chunfan Yang, Hongmei Zhao, Hongmei Su

**Affiliations:** 1 Beijing National Laboratory for Molecular Sciences (BNLMS), Institute of Chemistry, Chinese Academy of Sciences, Beijing 100190, China; qtx0521@iccas.ac.cn (T.Q.); hmzhao@iccas.ac.cn (H.Z.); 2 College of Chemistry, Beijing Normal University, Beijing 100875, China; kunhui@bnu.edu.cn (K.L.); yangchunfan@bnu.edu.cn (C.Y.); 3 University of Chinese Academy of Sciences, Beijing 100049, China

**Keywords:** Z-DNA, ZnTMPyP4, transient absorption spectroscopy, binding mode

## Abstract

Recognition of unusual left-handed Z-DNA by specific binding of small molecules is crucial for understanding biological functions in which this particular structure participates. Recent investigations indicate that zinc cationic porphyrin (ZnTMPyP4) is promising as a probe for recognizing Z-DNA due to its characteristic chiroptical properties upon binding with Z-DNA. However, binding mechanisms of the ZnTMPyP4/Z-DNA complex remain unclear. By employing time-resolved UV-visible absorption spectroscopy in conjunction with induced circular dichroism (ICD), UV-vis, and fluorescence measurements, we examined the binding interactions of ZnTMPyP4 towards B-DNA and Z-DNA. For the ZnTMPyP4/Z-DNA complex, two coexisting binding modes were identified as the electrostatic interaction between pyridyl groups and phosphate backbones, and the major groove binding by zinc(II) coordinating with the exposed guanine N_7_. The respective contribution of each mode is assessed, allowing a complete scenario of binding modes revealed for the ZnTMPyP4/Z-DNA. These interaction modes are quite different from those (intercalation and partial intercalation modes) for the ZnTMPyP4/B-DNA complex, thereby resulting in explicit differentiation between B-DNA and Z-DNA. Additionally, the binding interactions of planar TMPyP4 to DNA were also investigated as a comparison. It is shown that without available virtual orbitals to coordinate, TMPyP4 binds with Z-DNA solely in the intercalation mode, as with B-DNA, and the intercalation results in a structural transition from Z-DNA to B-ZNA. These results provide mechanistic insights for understanding ZnTMPyP4 as a probe of recognizing Z-DNA and afford a possible strategy for designing new porphyrin derivatives with available virtual orbitals for the discrimination of B-DNA and Z-DNA.

## 1. Introduction

DNA molecules are highly polymorphic and can form multiple conformations under different physiological conditions [1,2]. The most common structure of DNA is known as B-DNA, a right-handed double helix with the negatively charged deoxyribose-phosphate backbone outside and stacked base pairs inside. In some conditions, e.g., high ionic strengths, in the presence of highly charged cations or some small molecules, certain B-DNA sequences can transform to left-handed helical conformations called Z-DNA with the zig-zag backbone and the stacked base pairs partially exposed to the outside [2,3,4]. With the unique structure and higher energy, Z-DNA is less common than B-DNA [5,6]. Nevertheless, a considerable number of studies have demonstrated that Z-DNA is relevant to many biological processes; for example, it has been found that Z-DNA sequences participate in chromatin-dependent activation of the CSF1 (the human colony-stimulating factor 1 gene) promoter [7].

Differentiating B-DNA and Z-DNA is of fundamental importance for investigating the role played by Z-DNA in many biological processes [1]. Generally, recognition of Z-DNA is achieved by utilizing Z-DNA binding small molecules, which have particular binding modes to Z-DNA that are distinct from those to B-DNA, thus allowing the specific DNA form to be determined [8,9]. Although a large number of small molecules such as porphyrin molecules and its analogues, have been reported to bind with B-DNA and Z-DNA, only a few of them can recognize Z-DNA via detection of particular binding modes with Z-DNA [9,10,11,12]. This indicates that selecting an appropriate small molecule as a probe for Z-DNA requires a deep mechanistic understanding for the binding modes of small molecules to B-DNA and Z-DNA.

**Scheme 1:**
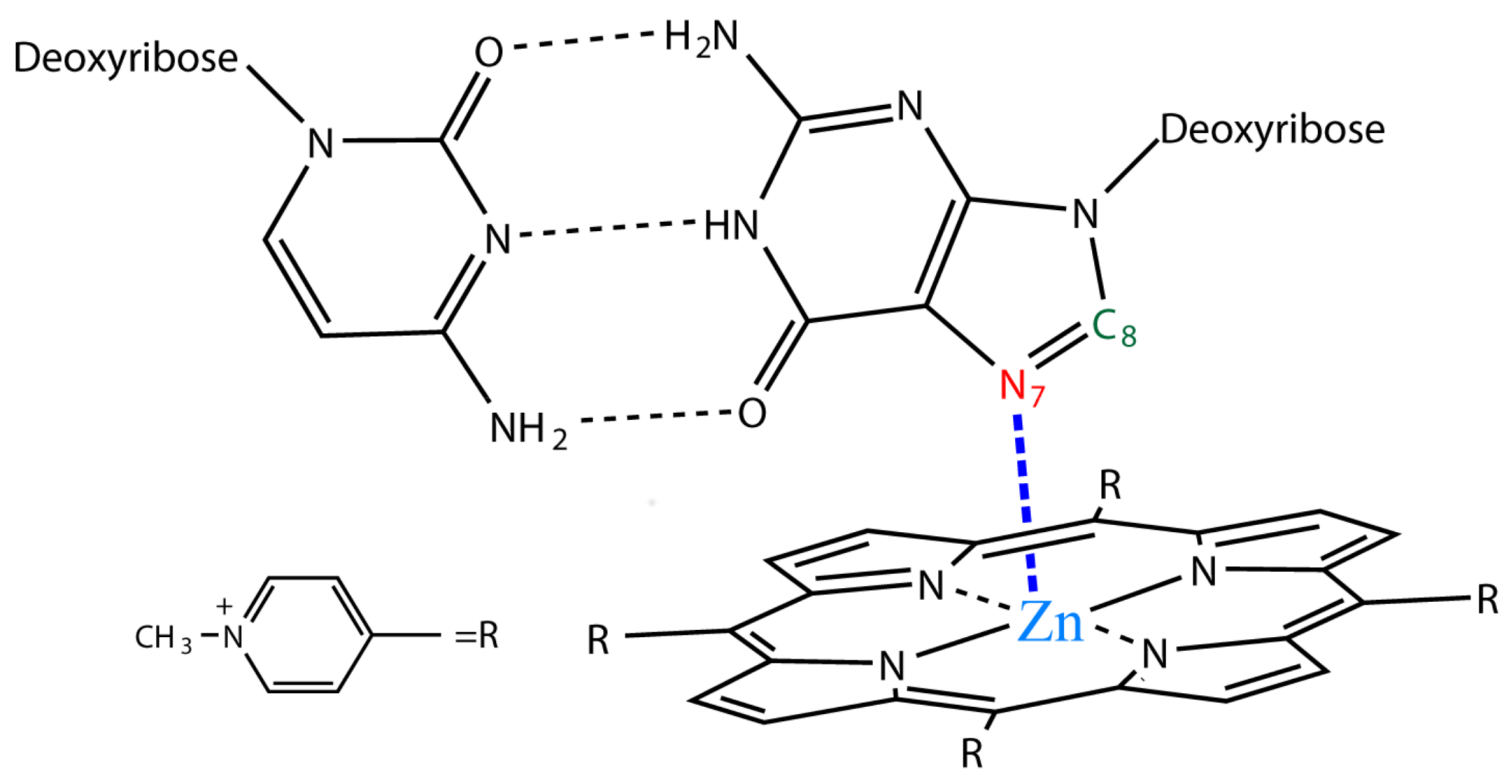
Schematic diagram for the central metal zinc(II) of ZnTMPyP4 coordinating with guanine N_7_ of Z-DNA.

As a derivative of meso-tetrakis[4-(*N*-methylpyridiumyl)]-21*H*,23*H*-porphyrin (TMPyP4), zinc(II) cationic porphyrin (ZnTMPyP4) has received much attention, because zinc(II) can additionally afford an axial coordination in the center of porphyrin, which may enable ZnTMPyP4 to become a promising probe for recognizing Z-DNA through different DNA binding behaviors. Balaz et al. investigated the binding of ZnTMPyP4 to B-DNA and Z-DNA (i.e., B-poly(dG-dC)_2_ and Z-poly(dG-dC)_2_) by using induced circular dichroism (ICD) spectroscopy [9,13], and obtained distinct ICD signals in the Soret region of ZnTMPyP4 with a small negative peak for B-DNA and a very intense bisignate curve for Z-DNA. They assumed that such a dramatic change in ICD spectra might be ascribed to different interaction modes between the binding of ZnTMPyP4 with B-DNA and Z-DNA. The small negative ICD peak suggests ZnTMPyP4 should be partially intercalated between GC pairs of B-DNA, whereas the intense bisignate curve reflects the electronic interaction between two bound ZnTMPyP4, and each ZnTMPyP4 may adopt its central metal zinc(II) to bind with guanine N_7_ of Z-DNA exposed externally (Scheme 1) [14].

Recently, Gong et al. studied the interactions of ZnTMPyP4 with Z-DNA (i.e., Z-poly(dG-dC)_2_) and B-DNA (i.e., B-poly(dG-dC)_2_) by means of linear dichroism (LD) spectroscopy [15]. They observed a strong positive LD peak in the Soret region for the ZnTMPyP4/Z-DNA complex, which is quite different from the LD spectrum of the ZnTMPyP4/B-DNA complex, which shows a very weak negative peak in the same region. By calculating angles of the B_x_ and B_y_ transitions of ZnTMPyP4 relative to the DNA helix axis, they proposed two possible ways for ZnTMPyP4 to bind with the major groove of Z-DNA: the molecular plane of porphyrin either parallel or perpendicular to the DNA helix axis.

To further understand the binding mechanisms underlying these spectroscopic changes, this work attempts to examine the binding of ZnTMPyP4 towards B-DNA and Z-DNA (B-poly(dG-dC)_2_ and Z-poly(dG-dC)_2_) respectively by employing time-resolved UV-visible absorption spectroscopy in conjunction with multiple steady-state spectroscopic techniques of ICD, UV-vis and fluorescence. The steady-state spectroscopic results first indicate a partial intercalation binding mode for the ZnTMPyP4/B-DNA complex and an external groove binding mode for ZnTMPyP4/Z-DNA complex. Further, time-resolved UV-visible absorption spectroscopy monitors the triplet-state decay kinetics of the bound ZnTMPyP4 with B-DNA and Z-DNA, and reveals that there are two coexisting binding modes for ZnTMPyP4 with each DNA. For ZnTMPyP4 interacting with B-DNA, the two lifetime components of 30.4 ± 0.1 μs (58%) and 11.9 ± 0.2 μs (42%) are separately assigned to the intercalation mode and partial intercalation mode, according to the degree of shielding of triplet states from oxygen quenching. For the binding of ZnTMPyP4 with Z-DNA, the shorter-lived component (3.4 ± 0.3 μs) arises from the electrostatic interaction between pyridyl groups of ZnTMPyP4 and phosphate backbones of Z-DNA, allowing two sides of the ZnTMPyP4 macrocycle to be almost fully exposed to oxygen quenching. The exposed guanine N_7_ atom in Z-DNA provides a site for axial coordination with the central zinc(II) of ZnTMPyP4. The longer-lived component (14.8 ± 0.2 μs) thereby corresponds to a major groove binding mode in which the central metal zinc(II) of ZnTMPyP4 coordinates with guanine N_7_ of Z-DNA. This assignment validates the existence of the axially coordinated binding between ZnTMPyP4 and Z-DNA. In addition, the binding interactions of planar TMPyP4 to DNA are also investigated. The comparison of TMPyP4 with ZnTMPyP4 reveals the key roles of the coordination ability of Zn(II) in altering the binding behavior of porphyrin. These results provide clear pictures of binding modes for ZnTMPyP4/DNA interactions and shed light on the mechanistic understanding of ZnTMPyP4 as a probe for recognizing the structural change form B-DNA and Z-DNA.

## 2. Results and Discussion

### 2.1. Characterizing the Formation of B-DNA and Z-DNA Conformations

**Figure 1:**
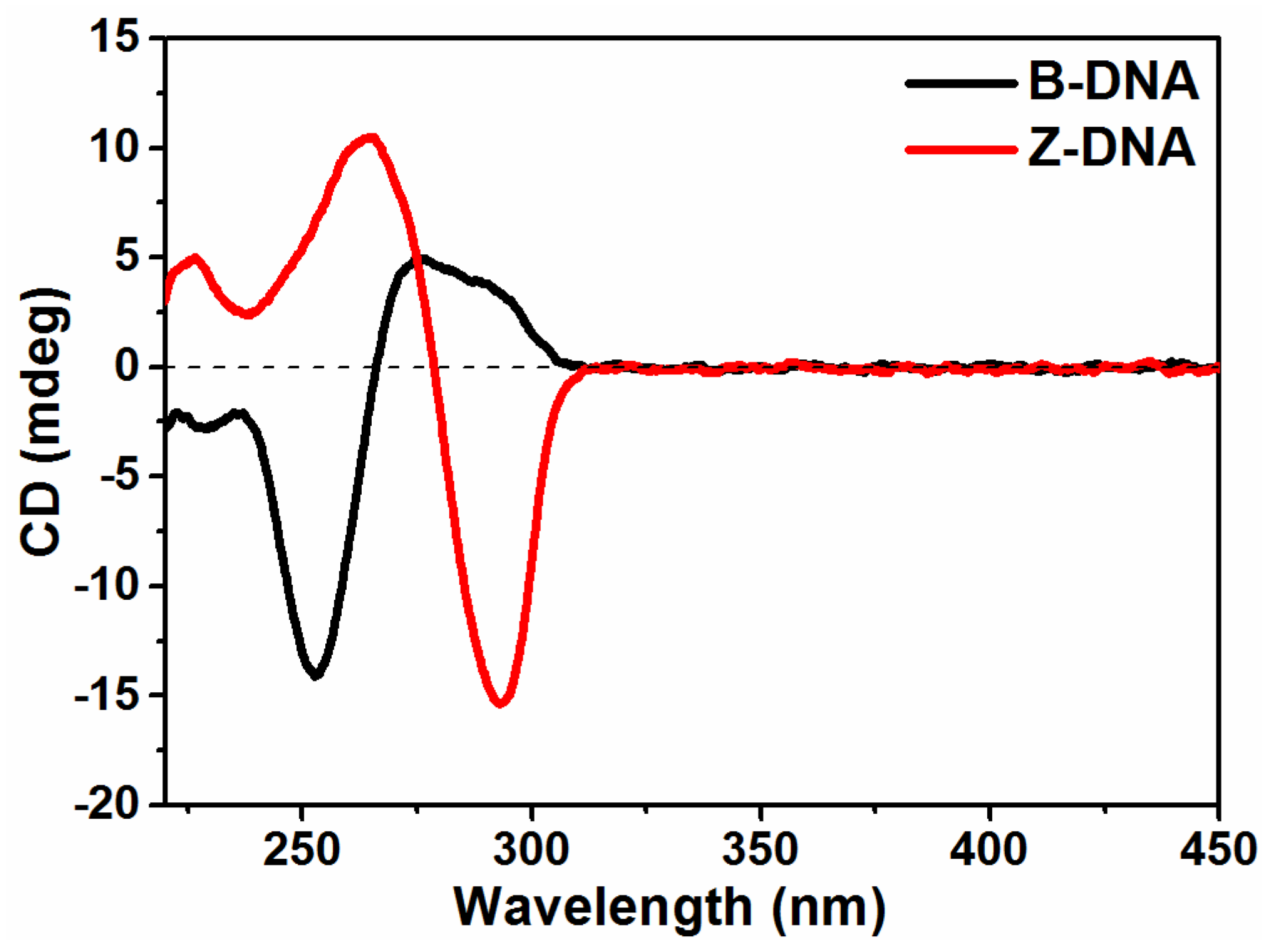
The CD spectra of B-DNA (50 μM) and Z-DNA (50 μM) in the presence of spermine (12 μM) in Na-cacodylate buffer (1 mM, pH = 7.0).

The alternating purine–pyrimidine sequence poly(dG-dC)_2_ (poly(deoxyguanylicdeoxycytidylic) acid; approximate average length in base pairs is 800) forms a typical B-DNA structure [9] and is subject to favorable transition to the Z-form conformation in the presence of micromolar concentrations of protonated spermine [13]. Figure 1 shows the Circular dichroism (CD) spectrum of B-poly(dG-dC)_2_ (denoted by B-DNA hereafter), which features a complex positive CD band centered at 274 nm and a negative CD band at 253 nm. Under the experimental conditions of spermine (12 μM) and Na-cacodylate buffer (1 mM, pH = 7.0), the CD spectrum presents a negative CD band at 293 nm and a positive CD band at 263 nm (Figure 1), which is characteristic for Z-DNA, indicating that the B-DNA has been successfully induced to the Z-poly(dG-dC)_2_ (denoted by Z-DNA hereafter).

### 2.2. Interaction of TMPyP4 with B-DNA and Z-DNA

**Figure 2:**
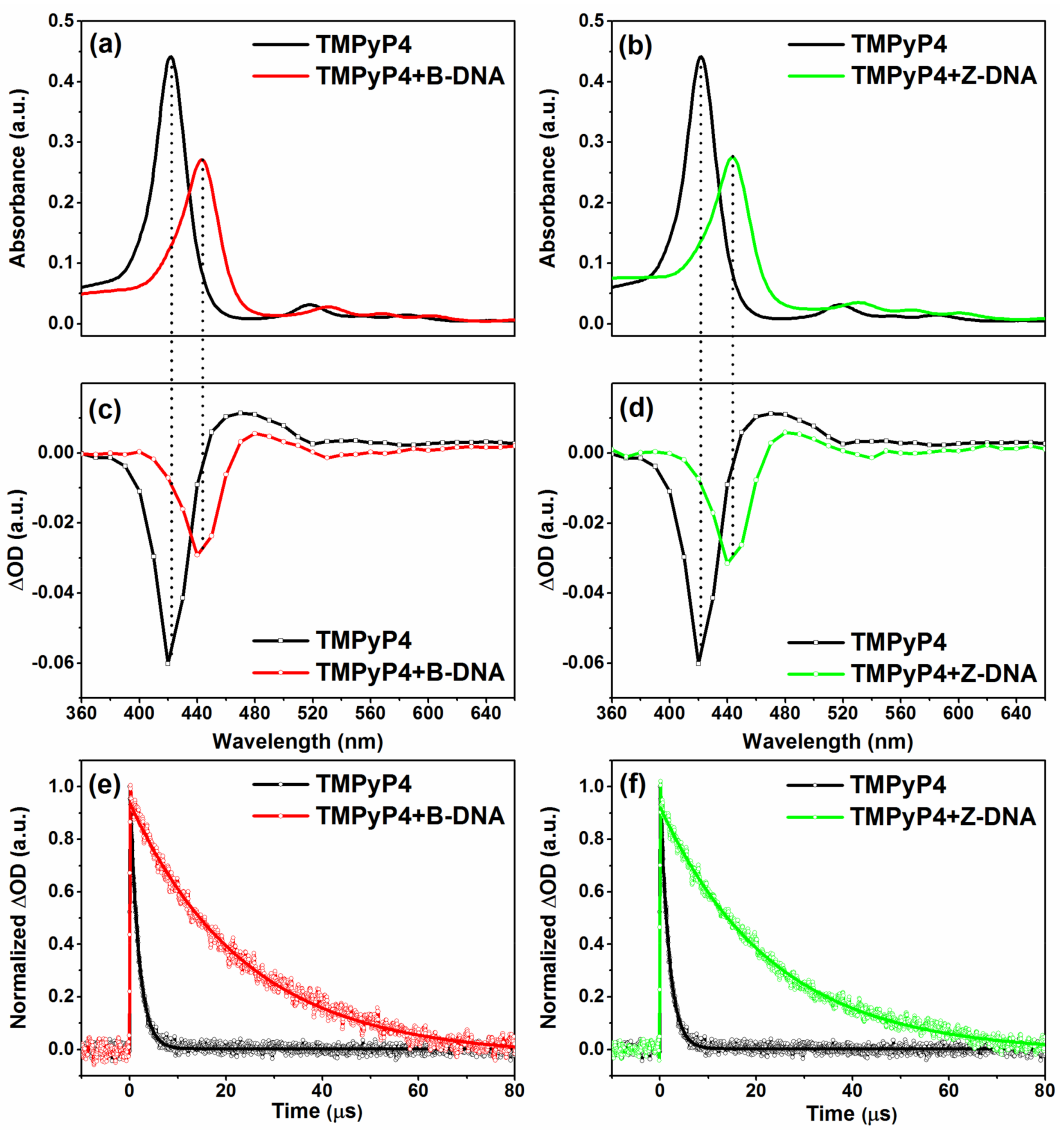
(**a**,**b**) Steady-state UV/Vis absorption spectra and (**c**,**d**) transient UV/Vis absorption spectra recorded instantly (50 ns) after laser flash photolysis upon 355 nm excitation for free TMPyP4 (2 μM) and its complexes with each of the two DNA (50 μM) in Na-cacodylate buffer (1 mM, pH = 7.0). Normalized triplet decay signals after laser flash photolysis of TMPyP4 (2 μM) upon 355 nm excitation in the absence (black) and presence of B-DNA (50 μM) (**e**) and Z-DNA (50 μM) (**f**). Fitted curves are shown by solid lines. The fits are all obtained from complete decay traces.

**Figure 3:**
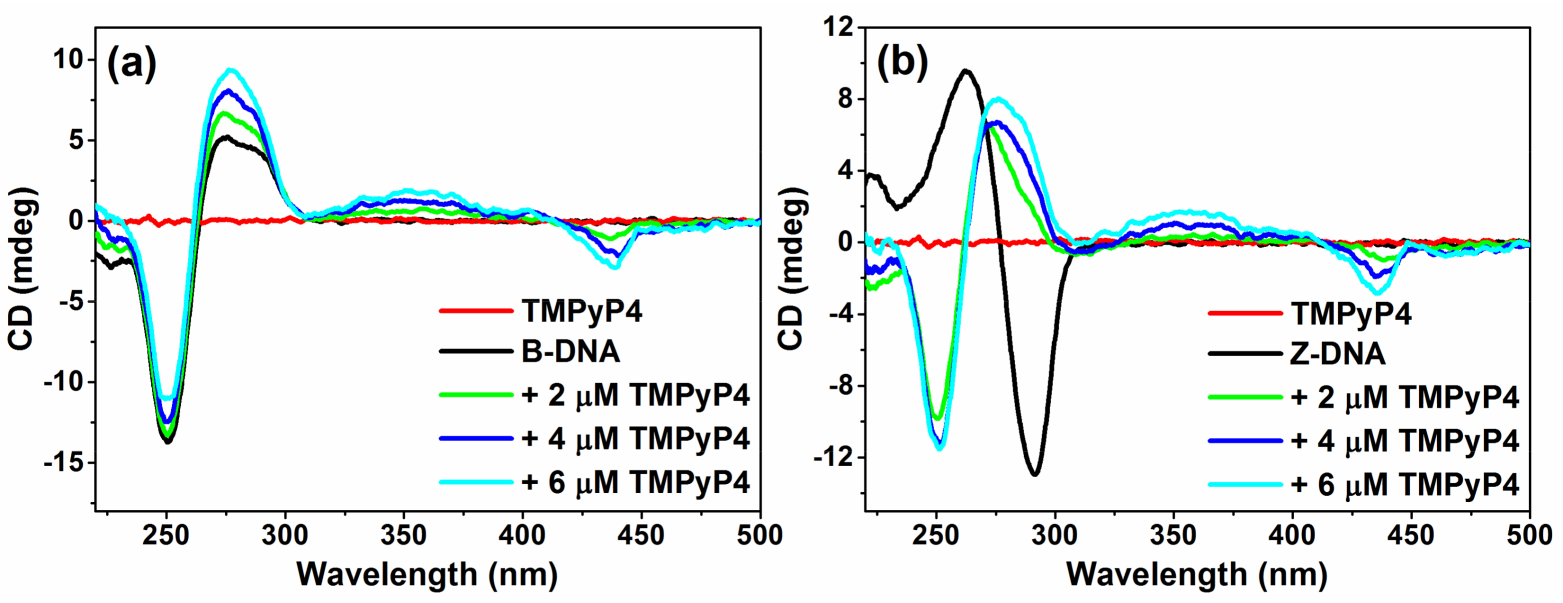
CD spectra of (**a**) B-DNA (50 μM) and (**b**) Z-DNA (50 μM) in the presence of different TMPyP4 concentrations from 2 to 6 μM in Na-cacodylate buffer (1 mM, pH = 7.0).

For comparison with ZnTMPyP4/DNA interactions, we first investigated the binding behaviors of planar porphyrin TMPyP4 with B-DNA and Z-DNA. Figure 2a shows the absorption spectra of TMPyP4 (2 μM) in the absence and presence of B-DNA (50 μM) in Na-cacodylate buffer. Compared to free TMPyP4, large hypochromic effects (*H* = 38%) and red-shifts (22 nm, from 422.0 to 444.0 nm) of the Soret band of TMPyP4 are observed in the presence of B-DNA, indicating the intercalative binding of TMPyP4 with B-DNA [16,17]. The ICD spectrum of TMPyP4 further confirms this intercalation binding mode, which displays an induced negative ICD band at 438 nm (−2.8 mdeg) in the presence of B-DNA, as shown in Figure 3a [16,18,19]. In addition, Figure S1a shows the splitting of the emission into two peaks and the decreased fluorescence intensity of the TMPyP4/B-DNA complex relative to free TMPyP4. This quenching may originate from a good π-π stacking between TMPyP4 and GC pair of B-DNA, implying the existence of the intercalation mode [20]. Interestingly, identical hypochromicity and bathochromic shift, similar negative ICD bands at 436 nm and fluorescence quenching also appear in the absorption, ICD and fluorescence spectra of TMPyP4 (Figure 2b, Figure 3b and Figure S1b) when B-DNA is replaced by Z-DNA. These spectroscopic results suggest that TMPyP4 also intercalates into Z-DNA, which is in good agreement with previous reports [10,11].

Second, transient absorption spectroscopy was performed to examine the binding of TMPyP4 to B-DNA and Z-DNA by monitoring the triplet state of TMPyP4. In the transient absorption spectrum of free TMPyP4, a positive peak at 470 nm due to triplet excited-state formation and the negative peak of ground-state depletion at 420 nm are observed, as shown in Figure 2c,d. Accompanying the hypochromicity, the transient absorption of the triplet state is red-shifted to 480 nm when the TMPyP4 is bound with either B-DNA or Z-DNA. Therefore, the triplet excited-state decay kinetics were monitored for free TMPyP4 at 470 nm and DNA/TMPyP4 complexes at 480 nm.

Figure 2e,f displays the triplet decay curves for TMPyP4 bound, respectively, with B-DNA and Z-DNA, in comparison with free TMPyP4. As can be seen, the triplet decay of free TMPyP4 follows a monoexponential law with a lifetime of 1.7 ± 0.02 μs, which matches early reports [21,22,23]. When TMPyP4 is bound with B-DNA or Z-DNA, the triplet decays become pronouncedly slower and exhibit first-order exponential laws separately with a lifetime of 27.8 ± 0.3 μs for B-DNA and a lifetime of 28.6 ± 0.2 μs for Z-DNA. Similar lifetimes might correspond to similar binding sites, since the triplet lifetime reflects the microenvironment of TMPyP4 bound with the duplex DNA, which could screen the triplet state from being quenched by oxygen molecules in bulk solution. According to the literature [24], the lifetime components of 27.8 ± 0.3 μs for B-DNA and 28.6 ± 0.2 μs for Z-DNA can both be assigned to the population in intercalation mode, which is consistent with the above steady-state experimental results.

From the steady-state and transient experimental results, it can be concluded that an identical binding mode (intercalation mode) is observed for TMPyP4 interacting with B-DNA or Z-DNA. Furthermore, intercalative TMPyP4 can cause the structural transition of Z-DNA to B-DNA, as evidenced by the disappearance of the marked CD peaks of Z-DNA and the appearance of the characteristic bands of B-DNA (Figure 3b) [10]. These results indicate that the planar TMPyP4 cannot serve as a probe for recognizing Z-DNA.

### 2.3. Interaction of ZnTMPyP4 with B-DNA and Z-DNA

#### 2.3.1. Interaction of ZnTMPyP4 with Right-Handed B-DNA

**Figure 4:**
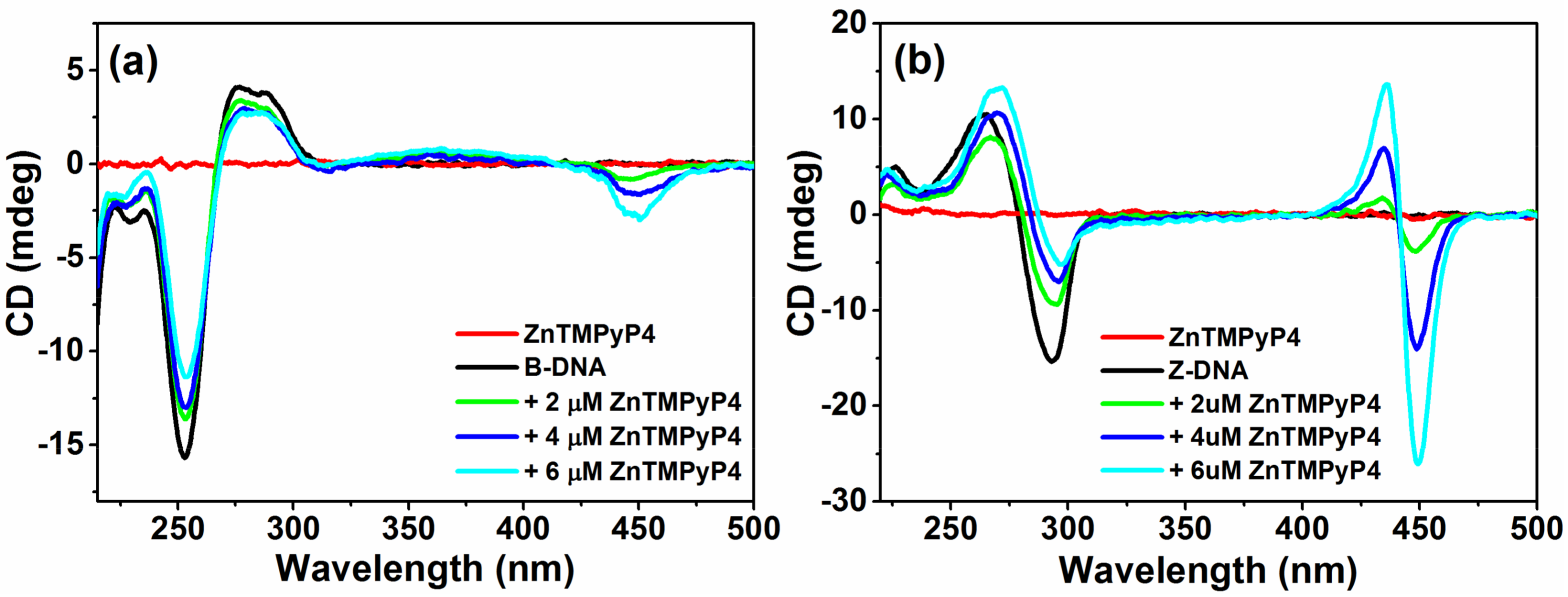
CD spectra of (**a**) B-DNA (50 μM) and (**b**) Z-DNA (50 μM) in the presence of different ZnTMPyP4 concentrations from 2 to 6 μM in Na-cacodylate buffer (1 mM, pH = 7.0).

**Figure 5:**
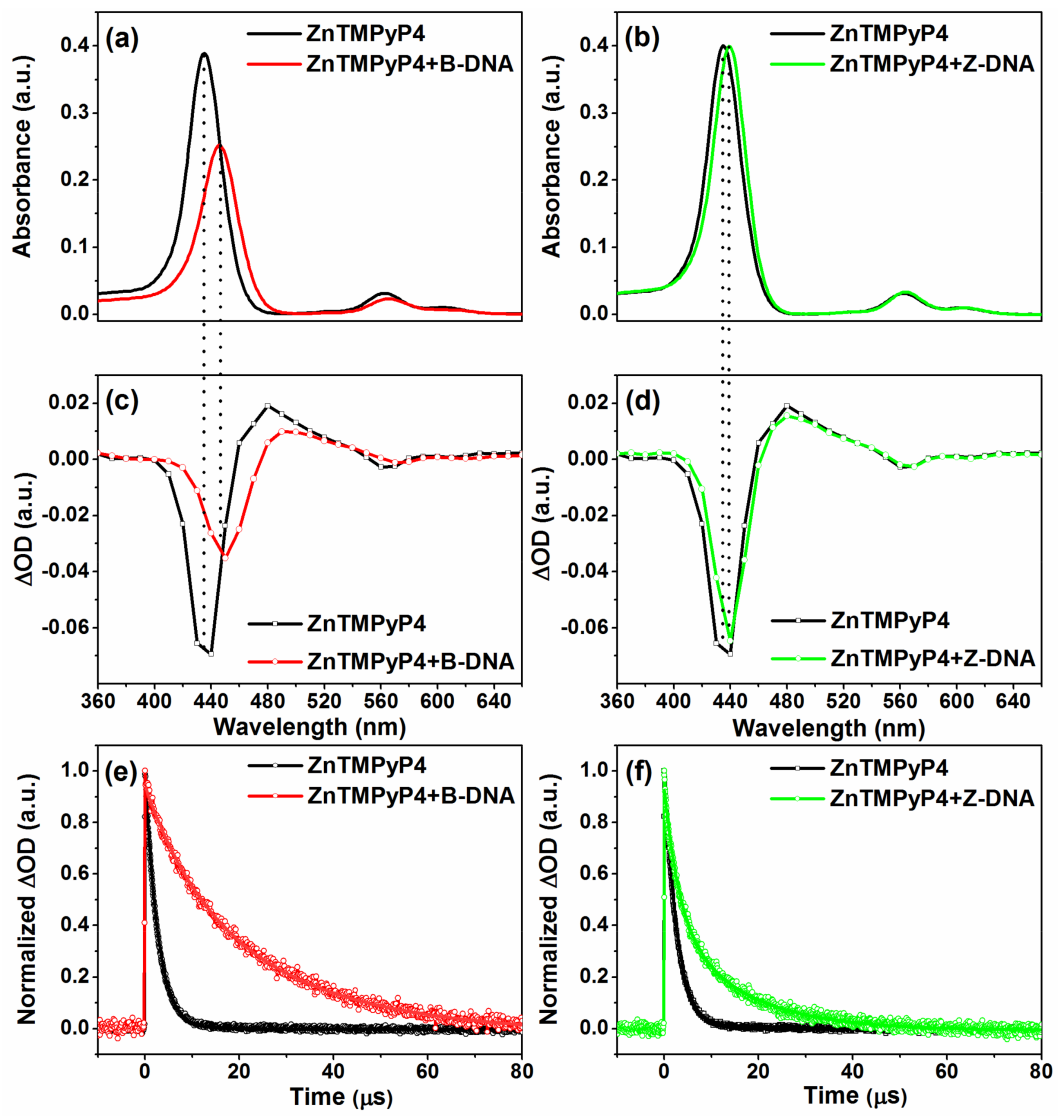
(**a**,**b**) Steady-state UV/Vis absorption spectra and (**c**,**d**) transient UV/Vis absorption spectra recorded instantly (50 ns) after laser flash photolysis upon 355 nm excitation for free ZnTMPyP4 (2 μM) and its complexes with each of the two DNA (50 μM)) in Na-cacodylate buffer (1 mM, pH = 7.0). Normalized triplet decay signals after laser flash photolysis of ZnTMPyP4 (2 μM) upon 355 nm excitation in the absence (black) and presence of B-DNA (50 μM) (**e**) and Z-DNA (50 μM) (**f**). Fitted curves are shown by solid lines. The fits are all obtained from complete decay traces.

Parallel spectroscopic studies were carried out for the binding of ZnTMPyP4 to B-DNA. Similar to TMPyP4, the ICD spectrum of ZnTMPyP4 displays an induced negative CD band at 448.0 nm (−2.7 mdeg) (Figure 4a), and the fluorescence spectrum shows reduced intensity (Figure S2a). However, the absorption spectrum of ZnTMPyP4 shows a similar hypochromic effect (*H* = 37%), but with a relative small bathochromic shift (10 nm) (Figure 5a) compared to TMPyP4. From these observations, an atypical intercalation mode—namely, partial intercalation—is suggested for the pentacoordinated ZnTMPyP4 with B-DNA, and this may be caused by the steric hindrance of the axially coordinated water molecule on zinc(II), which is in agreement with general knowledge of ZnTMPyP4 [13,23].

The interaction of ZnTMPyP4 with B-DNA was further examined using transient absorption spectroscopy by monitoring the triplet excited-state decay kinetics of ZnTMPyP4. As shown in Figure 5c, a positive peak (triplet excited-state formation) and negative peak (ground-state depletion) are observed at 480 nm and 440 nm in the transient absorption spectrum of free ZnTMPyP4. When ZnTMPyP4 binds to B-DNA, large hypochromicity and small redshifts (10 nm) are observed for both triplet excited-state formation and ground-state depletion, which is in accordance with the steady-state absorption spectra. Therefore, the triplet decay kinetics of free ZnTMPyP4 and the ZnTMPyP4/B-DNA complex were measured at 480 nm and at 490 nm, respectively.

As shown in Figure 5e, a monoexponential decay behavior with a triplet lifetime of 2.6 ± 0.01 μs is exhibited for free ZnTMPyP4, and this inherent triplet lifetime is longer than that of TMPyP4. In contrast, the triplet state decay kinetics of the ZnTMPyP4/B-DNA complex becomes much slower and can be well fitted by two exponential components with lifetimes of 11.9 ± 0.2 μs and 30.4 ± 0.1 μs (Figure 5e, Table 1). As mentioned above, the triplet state lifetime reflects the degree of shielding of the triplet ligand from oxygen quenching; thus, the biexponential decay for the bound ZnTMPyP4 strongly implies the simultaneous existence of two binding modes. One of the two coexisting binding modes should be the partial intercalation mode, which is also suggested by the steady-state experiments. Since the intercalation of ZnTMPyP4 between GC base pairs of the duplex was characterized in previous work [20], and the axial water was assumed to be released by gaining enough energy during its intercalation process, the other binding mode may possibly be the intercalation mode. Obviously, the intercalation mode much better protects the porphyrin macrocycle from molecular oxygen access, which quenches the triplet state, than the partial intercalation mode. The currently observed longer lifetime component of 30.4 ± 0.1 μs should thus correspond to the population bound in the intercalation mode, whereas the shorter lifetime component of 11.9 ± 0.2 μs is assigned to the population bound in the partial intercalation mode. The 30.4 ± 0.1 μs lifetime is comparable with the intercalative mode of TMPyP4 (27.8 ± 0.3 μs or 28.6 ± 0.2 μs) in poly(dG-dC)_2_, indicating the reasonability of this assignment.

In addition, the triplet reporter method also assesses the contribution of different binding modes. With the biexponential fitting, the pre-exponential factors obtained for the two lifetime components actually correspond to the respective percentages of the binding modes, and these values are also listed in Table 1. From Table 1, it is shown that intercalation is the main binding mode for nearly 58%, while the partial intercalation mode accounts for ~42%.

#### 2.3.2. Interaction of ZnTMPyP4 with Left-Handed Z-DNA

**Scheme 2:**
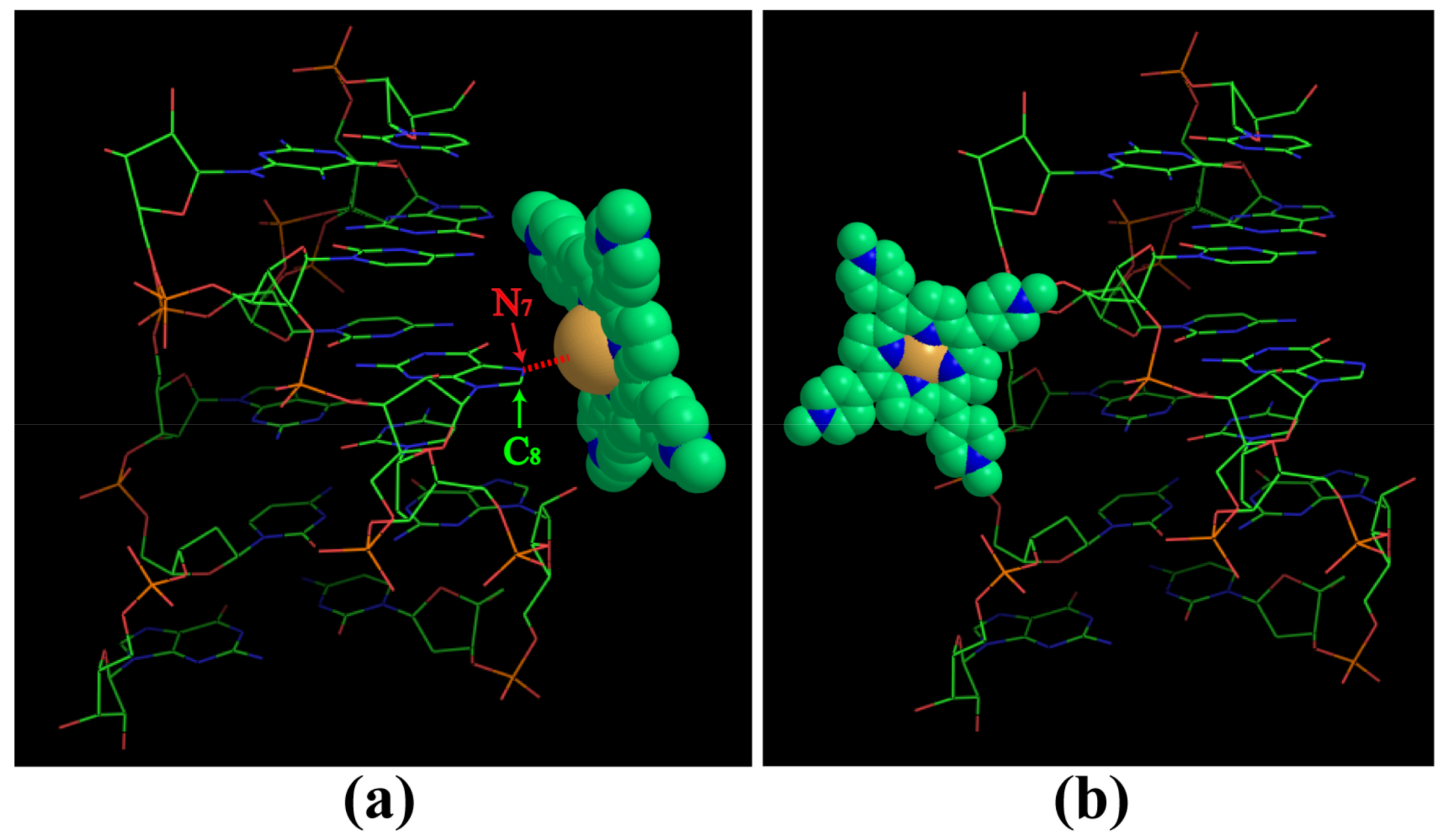
Depiction of the possible binding modes of ZnTMPyP4 to the Z-DNA: (**a**) the major groove coordination binding mode; (**b**) the electrostatic interaction binding mode. (Crystal structure of the Z-DNA with about half a turn was extracted from RCSB, PDB entry: 5EBI [26]).

The binding of ZnTMPyP4 with Z-DNA was further examined, and is different from that with B-DNA, interestingly. As shown in Figure 5b, addition of ZnTMPyP4 to the spermine-induced Z-form of poly(dG–dC)_2_ results in a bathochromic shift (Δ*λ* = 4 nm, from 435 to 439 nm) and no hypochromicity of the Soret band, which is quite different from the absorption spectrum of ZnTMPyP4/B-DNA, which displays large hypochromic effects (*H* = 37%) and red-shifts (10 nm). Normally, for the interaction of porphyrin with duplexes, the intercalation mode results in a strong bathochromic shift (≥15 nm) and marked hypochromicity (*H* ≥ 35%) in the Soret band [16,17], whereas the external groove binding mode exhibits a smaller bathochromic shift (≤8 nm) and weaker hypochromicity (*H* ≤ 10%) [16,19]. Therefore, in the current work, such a small red-shift and absence of hypochromicity suggest an external groove binding mode for the ZnTMPyP4/Z-DNA complex (Scheme 2), instead of the partial intercalation and intercalation modes that are detected for the ZnTMPyP4/B-DNA complex.

The different binding behavior can also be seen from the fluorescence spectrum of the ZnTMPyP4/Z-DNA complex, which is shown in Figure S2b. Unlike TMPyP4, the fluorescence of ZnTMPyP4 is not quenched upon binding to Z-DNA, and the emission at 635 nm is obviously blue-shifted to 627 nm. This is clearly indicative of the absence of π-π interactions between GC base pair and ZnTMPyP4 core and the existence of an external groove binding mode in which guanine N_7_ atom of Z-DNA is available for coordination with Zn(II), as concluded in previous fluorescence studies [9].

Figure 4b shows the CD spectrum of ZnTMPyP4 in the presence of Z-DNA, which reveals a bisignate signal in the Soret region with a negative CD band at 450 nm (−26 mdeg) and a positive CD band at 436 nm (+14 mdeg). As discussed by previous reports, the induced bisignate CD signals may originate from long-distance through-space, electric dipole–dipole, and exciton coupling of the two ZnTMPyP4 chromophores [9,25], which might adopt a groove binding mode with substitution of the axial water molecule in ZnTMPyP4 by guanine N_7_ of Z-DNA.

To further reveal the binding modes of ZnTMPyP4 with Z-DNA, transient absorption spectroscopy measurements were performed. In the transient absorption spectrum of the bound ZnTMPyP4 with Z-DNA, no hypochromicity and slight redshifts are observed for triplet state formation and ground-state depletion (Figure 5d). The triplet state decay kinetics of ZnTMPyP4/Z-DNA complex was measured at 480 nm. Figure 5f shows its triplet decay behavior, which is obviously distinct from that of the ZnTMPyP4/B-DNA complex. The ZnTMPyP4 triplet state follows a second-order exponential decay with two lifetime components of 3.4 ± 0.3 μs and 14.8 ± 0.2 μs, which suggests the existence of two coexisting binding modes with their respective percentages of 53% and 47%.

The long lifetime of 14.8 ± 0.2 μs is much shorter than the typical intercalative lifetime (~30 μs) [24], which first excludes the possibility of the intercalation mode for ZnTMPyP4/Z-DNA complex. The possibility of a partial intercalation mode for ZnTMPyP4/Z-DNA complex can be also ruled out, according to the structural features of Z-DNA. Previous structural inspections of Z-DNA have indicated that the alternate *anti* and *syn* conformation of nucleobases leads to exposure of guanine atoms N_7_ and C_8_, which are both shielded in the B form [2]. In Z-DNA, the nitrogen atom N_7_ is available for coordination with transition-metal ions, as shown previously by IR study [27,28], and thus can provide a site for axial coordination with the central Zn(II) of ZnTMPyP4 (Scheme 2a). It is well known that the nitrogen atom N is less electronegative and more easily renders electron pairs than the oxygen atom O, resulting in its stronger coordination ability. Therefore, the central Zn(II) of ZnTMPyP4 should more readily coordinate with the guanine atom N_7_ of Z-DNA than the water molecule in the solution, which facilitates the coordination binding of ZnTMPyP4 with the guanine N_7_ of Z-DNA, and rules out the possibility of partial intercalation binding.

Additionally, it should also be noted that the minor groove of Z-DNA is narrow and deep, and spermine is located here [29,30], preventing the binding of the ZnTMPyP4 in the minor groove. In this case, the most plausible binding mode for the long lifetime of 14.8 ± 0.2 μs is coordination binding in the major groove with substitution of the axial water molecule in ZnTMPyP4 by guanine N_7_ of Z-DNA, as shown in Scheme 2a. In the geometry associated with this binding mode, one side of the prophyrin macrocycle faces the major groove of Z-DNA, while the other side is exposed to the solution. It follows that only one side of ZnTMPyP4 can receive efficient protection from oxygen access, leading to a moderate triplet state lifetime. This assignment of the coordination binding in the major groove is corroborated by the CD research results of the ZnTMPyP4/Z-DNA complex, in which induced bisignate CD signals due to the electronic coupling of the two ZnTMPyP4 chromophores are observed.

As for the short lifetime of 3.4 ± 0.3 μs of Z-DNA/ZnTMPyP4 complex, it is close to the typical triplet lifetime corresponding to electrostatic interaction binding mode, which was obtained for ZnTMPyP4 in B-poly(dG-dC)_2_ (3.5 μs) at high ionic strength by Chirvony, V.S. et al. [20]. Consequently, the 3.4 ± 0.3 μs lifetime component can be assigned to the population in the electrostatic interaction binding mode. In this binding geometry (Scheme 2b), ZnTMPyP4 is bound electrostatically by the positively charged pyridyl groups to negatively charged phosphate backbones on the outside with minimal interaction between the ZnTMPyP4 macrocycle and Z-DNA, allowing the exposure of two sides of ZnTMPyP4 to oxygen quenching and thus causing a short triplet state lifetime of ~3.4 μs. This assignment is supported by LD study, which proposed that the ZnTMPyP4 molecule also binds across the groove by electrostatic interaction, with the molecular plane of porphyrin perpendicular to the DNA helix axis [15].

Notably, the short lifetime of 3.4 ± 0.3 μs of ZnTMPyP4/Z-DNA complex is only 31% longer than that of free ZnTMPyP4 (2.6 ± 0.01 μs). Should this lifetime component also be ascribed to the unbound ZnTMPyP4 in the solution? To scrutinize this possibility, additional CD spectra of ZnTMPyP4 were measured at different [ZnTMPyP4]/[Z-DNA] molar ratios ranging from 0.04 to 0.16. As shown in Figure 4b, the intensity of the bisignate peak at the soret region increases with increasing [ZnTMPyP4]/[Z-DNA] ratio. The saturation of the ICD signal is observed at the [ZnTMPyP4]/[Z-DNA] of 0.12 with the ICD of −26 mdeg (Figure S3). In our triplet state experiment, the concentration of the Z-poly(dG–dC)_2_ (per base pair) is 50 μM and the concentration of the ZnTMPyP4 is 2 μM. Therefore, the [ZnTMPyP4]/[Z-DNA] ratio used in this work is 0.04, which is much lower than the binding saturation ratio 0.12. This indicates that each ZnTMPyP4 molecule is bound to the Z-DNA, thus ruling out the possibility that the short lifetime component of 3.4 ± 0.3 μs is caused by free ZnTMPyP4.

In brief, the triplet state kinetics results, together with the steady-state experimental results, reveal the simultaneous existence of major groove binding by forming N_7_-Zn coordination bond and an electrostatic binding for the ZnTMPyP4/Z-DNA complex, which are quite different from the case of the ZnTMPyP4/B-DNA complex. In addition, compared with planar TMPyP4, ZnTMPyP4 provides a virtual orbital to coordinate with the exposed guanine N_7_ atom of Z-DNA, leading to the unusual binding mode of ZnTMPyP4 distinguishing Z-DNA from B-DNA. These results not only provide mechanistic insights for ZnTMPyP4 acting as a probe for recognizing Z-DNA, but also afford a possible strategy for designing new porphyrin derivatives with available virtual orbitals for the discrimination of B-DNA and Z-DNA.

## 3. Materials and Methods

### 3.1. Materials

The porphyrin derivative meso-tetrakis[4-(*N*-methylpyridiumyl)]-21*H*,23*H*-porphyrin (TMPyP4) in the form of tetra-p-tosylate salt was purchased from Tokyo Chemical Industry (TCI, Tokyo, Japan) and used as received. Zinc(II)-meso-tetrakis(4-(*N*-methylpyridiumyl))-porphyrin (ZnTMPyP4) was purchased from Frontier Scientific (Logan, UT, USA) and used without any further purification. Poly(deoxyguanylic–deoxycytidylic) acid sodium salt (poly(dG–dC)_2_), sodium cacodylate, sodium chloride, spermine tetrahydrochloride (C_10_H_26_N_4_·4HCl) were purchased from Sigma-Aldrich (St. Louis, MO, USA). Deionized water was obtained from a Milli-Q system with a resistivity of 18.2 MΩ·cm. DNA samples were prepared in a sodium cacodylate buffer (1 mM, pH 7.0), annealed at 70 °C for 20 min, and then cooled to 10 °C at 1 °C/min, and kept at 4 °C for 12 h. The concentration of the poly(dG–dC)_2_ (per base pair) was quantified by UV-vis spectroscopy using the extinction coefficient *ε* = 8.8 × 10^3^ M^−1^·cm^−1^ at 260 nm [13]. The porphyrin stock solutions were prepared in a sodium cacodylate buffer (1 mM, pH 7.0), and the concentration was determined by UV-vis spectroscopy using the following extinction coefficients *ε* = 2.26 × 10^5^ M^−1^·cm^−1^ at 424 nm for TMPyP4 [22] and *ε* = 2.04 × 10^5^ M^−1^·cm^−1^ at 437 nm for ZnTMPyP4 [16].

### 3.2. Steady-State Spectroscopy

CD spectra were recorded at room temperature on a Jasco J-815 spectropolarimeter (JASCO, Oklahoma City, OK, USA). Each sample was collected from 550 to 200 nm at a scan speed of 200 nm/min with a response time of 0.5 s in a 1 cm quartz cell. The reported spectroscopy was the average of three scans. The spectrum from a blank sample containing only buffer was used as the background that was subtracted from the averaged data. Steady-state absorption spectroscopy was recorded with a UV-vis spectrometer (model U-3010, Hitachi, Tokyo, Japan). Quartz cuvettes of 1 cm path length were used for all absorption measurement. Fluorescence spectroscopy was measured with a fluorescence spectrometer (F4600, Hitachi). All samples were excited at 355 nm.

### 3.3. Laser Flash Photolysis

Transient UV-visible spectroscopy and triplet state kinetics were measured by a nanosecond time-resolved laser flash photolysis (LFP) setup that has been described previously [22]. Briefly, the instrument comprises an Edinburgh LP920 spectrometer (Edinburgh Instrument Ltd., Livingstone, UK) combined with an Nd:YAG laser (Surelite II, Continuum Inc., Christiansburg, VI, USA). The excitation wavelength is a 355 nm laser pulse from Q-switched Nd:YAG laser (1 Hz, fwhm ≈ 7 ns, 10 mJ/pulse). The analyzing light is from a 450 W pulsed xenon lamp. A monochromator equipped with a photomultiplier for collecting the spectroscopy range from 350 to 700 nm was used to analyze transient absorption spectroscopy. The signals from the photomultiplier were displayed and recorded as a function of time on a 100 MHz (1.25 Gs/s sampling rate) oscilloscope (Tektronix, Beaverton, OR, USA, TDS 3012B), and the data were transferred to a personal computer. Data were analyzed by the online software of the LP920 spectrophotometer. The fitting quality was judged by weighted residuals and reduced *χ*^2^ value. Sample solutions were freshly prepared for each measurement.

### 3.4. Structural Transition from B-DNA to Z-DNA

Micromolar concentration of spermine (a fully protonated tetraamine at pH = 7.0) was used to induce the B-DNA to Z-DNA transition in vitro. Z-DNA was formed by incubating at 60 °C with 12 μM spermine for 10 min, then slowly cooled down to room temperature. The successful transition from B-DNA to Z-DNA was ensured by CD spectroscopic results showing the characteristics of left-handed Z-DNA: negative CD bands at 293 nm, and a positive CD band at 263 nm [13].

## 4. Conclusions

In this work, we comprehensively studied the binding interactions of ZnTMPyP4 with two different duplex DNA, B-DNA and Z-DNA, by combining transient UV-vis absorption spectroscopy with steady-state measurements (UV-vis, fluorescence, and ICD). For the ZnTMPyP4/B-DNA complex, it is found that the partial intercalation and intercalation modes through π-π stacking of ZnTMPyP4 macrocycle with GC base pairs coexist. In contrast to this type of π-π stacking interaction mode, ZnTMPyP4 binds with Z-DNA by means of another two interaction modes, i.e., the electrostatic interaction between pyridyl groups and phosphate backbones, and major groove binding by Zn(II) coordinating with the exposed guanine N_7_. The unusual binding behaviors of ZnTMPyP4 with Z-DNA thus make it possible to utilize ZnTMPyP4 as a probe for discerning Z-DNA from B-DNA. In addition, we also investigated the binding of planar TMPyP4 to B-DNA and Z-DNA as a comparison. It is shown that without available virtual orbitals to coordinate, TMPyP4 binds with Z-DNA solely in the intercalation mode, which is identical to B-DNA. The intercalation of TMPyP4 results in structural transition from Z-DNA to B-ZNA. These results elaborate the binding mechanisms of ZnTMPyP4 with Z-DNA in comparison with B-DNA and reveal the key roles of the coordination ability of the Zn(II) cation in altering the binding behavior of porphyrin, thus providing valuable guidance for the design and selection of molecular probes in recognizing the important Z-DNA structure.

## Figures and Tables

**Table 1:** Triplet decay lifetimes of TMPyP4 and ZnTMPyP4 in air-saturated solution and in their complexes with B-DNA and Z-DNA. Pre-exponential factors of the two lifetime components in the biexponential fitting $I=I_{0}+A_{1}e^{-t/\tau_{1}}+A_{2}e^{-t/\tau_{2}}$ yield the respective percentages of two binding modes (values shown in brackets).

Sample	*τ*_1_ (μs)	*τ*_2_ (μs)
TMPyP4	1.7 ± 0.02	
TMPyP4+B-DNA	27.8 ± 0.3	
TMPyP4+Z-DNA	28.6 ± 0.2	
ZnTMPyP4	2.6 ± 0.01	
ZnTMPyP4+B-DNA	11.9 ± 0.2 (42%)	30.4 ± 0.1 (58%)
ZnTMPyP4+Z-DNA	3.4 ± 0.3 (53%)	14.8 ± 0.2 (47%)

## Supplementary Materials

Supplementary materials can be found at http://www.mdpi.com/1422-0067/19/4/1071/s1.
