# Supplementary material for: Binding Interactions of Zinc Cationic Porphyrin with Duplex DNA: From B-DNA to Z-DNA
Source: Int J Mol Sci. 2018 Apr 4;19(4):1071. doi: 10.3390/ijms19041071 (PMC5979451; doi:10.3390/ijms19041071)
Supplement: Supplementary file 1 [file ijms-19-01071-s001.pdf]

Supplementary

Binding Interactions of Zinc Cationic Porphyrin with Duplex DNA: From B-DNA to Z-DNA

Tingxiao Qin [1,2,3], Kunhui Liu [3], Di Song [1,*], Chunfan Yang [3], Hongmei Zhao [1] and Hongmei Su [3,*]

[1] Beijing National Laboratory for Molecular Sciences (BNLMS), Institute of Chemistry, Chinese Academy of Sciences, Beijing 100190, China; qtx0521@iccas.ac.cn (T.Q.); hmzhao@iccas.ac.cn (H.Z.)

[2] College of Chemistry, Beijing Normal University, Beijing 100875, China; kunhui@bnu.edu.cn (K.L.); yangchunfan@bnu.edu.cn (C.Y.)

[3] University of Chinese Academy of Sciences, Beijing 100049, China

* Correspondence: songdi@iccas.ac.cn (D.S.); hongmei@bnu.edu.cn (H.S.); Tel.: +86-10-6255-2723 (D.S.)

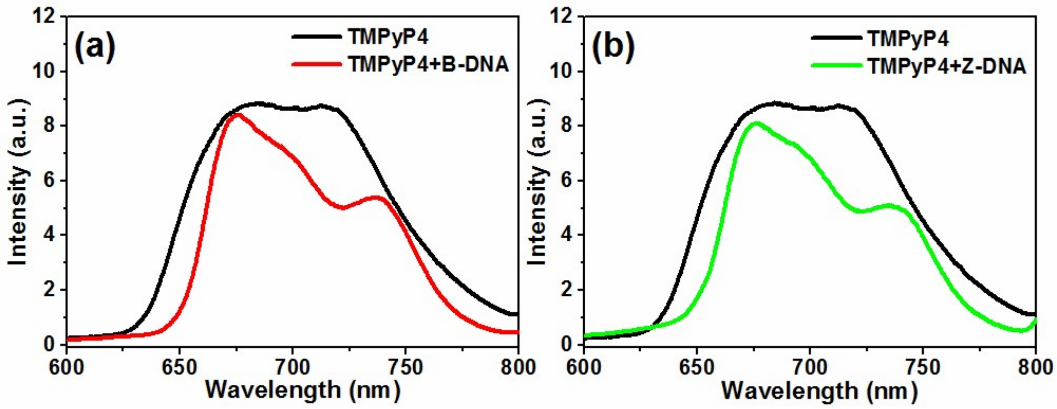

Figure S1. Fluorescence spectra of TMPyP4 in the absence and presence of B-DNA and Z-DNA of poly(dG–dC)$_2$ (50 μM) in Na-cacodylate buffer (1 mM, pH = 7.0).

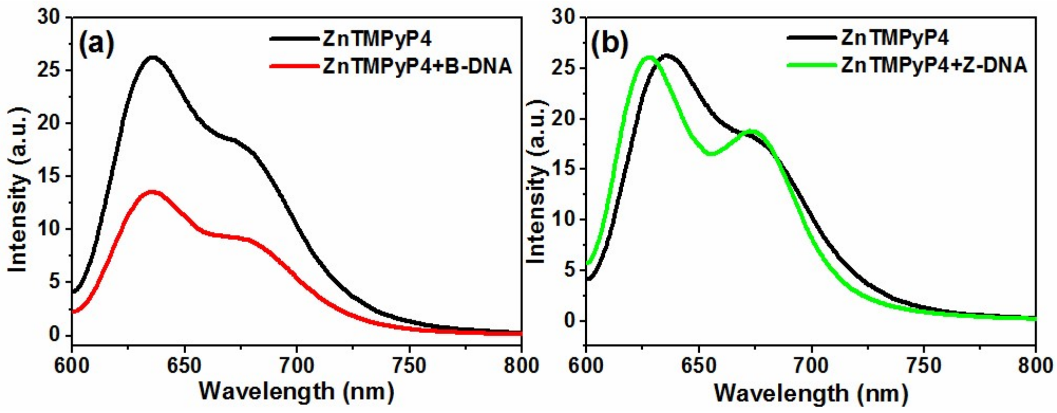

Figure S2. Fluorescence spectra of ZnTMPyP4 in the absence and presence of B-DNA (a) and Z-DNA (b) of poly(dG–dC)$_2$ (50 μM) in Na-cacodylate buffer (1 mM, pH = 7.0).

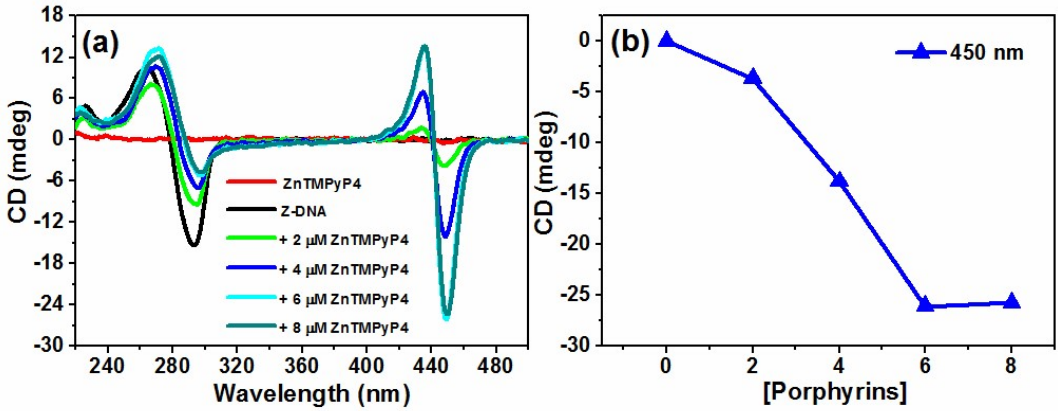

Figure S3. (a) Z-DNA of poly(dG–dC)$_2$ (50 μM) in the presence of different ZnTMPyP4 concentrations from 0 μM to 8 μM in Na-cacodylate buffer (1 mM, pH = 7.0). (b) CD signal as a function of porphyrin concentration at 450 nm.
